# Evaluation of vaccine effectiveness against SARS‐CoV‐2 variants by serological epitope profiling

**DOI:** 10.1002/ctm2.1182

**Published:** 2023-01-18

**Authors:** Cheng Li, Zhenlin Yang, Yanling Wu, Tianlei Ying

**Affiliations:** ^1^ MOE/NHC/CAMS Key Laboratory of Medical Molecular Virology Shanghai Institute of Infectious Disease and Biosecurity Shanghai Engineering Research Center for Synthetic Immunology School of Basic Medical Sciences Fudan University Shanghai China; ^2^ Shanghai Key Laboratory of Lung Inflammation and Injury Department of Pulmonary Medicine Zhongshan Hospital Fudan University Shanghai China

1

Over 30 vaccines for COVID‐19 have been approved worldwide; however, vaccine effectiveness has been severely compromised due to frequent viral mutations, especially against the SARS‐CoV‐2 Omicron variant and its emerging sublineages. In general, the generation of virus‐specific antibodies is considered crucial for protection against the viral infections, and the viral protein binding and viral neutralization are the most prevalent serological assays to assess the level of humoral immune response in vaccine candidates. However, since the emergence of new variants, the reduction of serum neutralizing potency was widely reported, making the current serological tests difficult to predict the protective potency against the continuously emerging mutants. Furthermore, it is always time consuming and labour intensive to discretely operate antibody neutralizing assay on each variant side by side. Therefore, a serological test for determining the potency and breadth of antiviral antibodies in seropositive individuals would be used to predict vaccine efficacy or reinfection risk in recovered COVID‐19 patients.

The receptor‐binding domain (RBD) of SARS‐CoV‐2, responsible for angiotensin‐converting enzyme 2 (ACE2) binding, is a critical target of neutralizing antibodies. Although several RBD‐specific monoclonal antibodies (mAbs) against SARS‐CoV‐2 have been approved by FDA under emergency use authorizations (EUAs), including bebtelovimab, casirivimab/imdevimab, tixagevimab/cilgavimab, sotrovimab and so forth, most of them were suspended due to the viral escape. As a result of the frequent mutations in RBD, numerous studies have discovered that the neutralizing potency of vaccinated sera and RBD‐specific antibodies was attenuated remarkably by Omicron lineage.^1^, ^2^ In fact, the epitopes targeted by antibodies have been widely attracted to researchers, and many studies identified different epitopes of neutralizing antibodies isolated from convalescents. These studies also explored the shared principles of viral escape from antibodies targeting different epitopes. Among them, a study of a big panel of 186 RBD‐directed mAbs^3^ classified antibodies into three clusters: antibodies that bind to (a) the receptor‐binding motif (RBM), (b) the outer face and (c) the inner face of RBD according to the orientation of the binding surface in the spike trimer. The escape of antibodies is found to be closely related to the particular mutations of the binding epitope. As a result, neutralization of RBM antibodies was affected the most by current circulating mutations, followed by that of the outer surface antibodies, and barely in antibody recognizing the inner face, suggesting that the inner face is more resilient to viral escape. Consequently, it is important to estimate the proportion of different antibody types in a certain seropositive individual. However, the isolation of mAbs from patients or vaccinated individuals is a tedious task, and some specific antibody populations can be lost during this process. Furthermore, in some studies, it was found that the escape maps of polyclonal plasma were often different from those of mAbs isolated from the same individual. Therefore, an ideal epitope profiling assay should be performed using polyclonal serum instead of isolated mAbs, which allows for high‐throughput, cost‐effective and accurate analysis of antibody composition in a large number of samples.

In a recent paper in *Cell*,^4^ we and others established an approach termed as ‘serological competition assay’, which integrates diverse facets of antibody analysis including the levels, potency and epitopes of antibodies that are critical to evaluate humoral immune response. First, polyclonal plasmas from inactivated vaccine recipients were used to test the binding and neutralizing activity against Omicron according to conventional methods. Next, in order to verify the correlation between antibody epitopes and antibody activity to Omicron, an approach was developed using biolayer interferometry assay (BLI) and aimed to detect the levels of RBM antibodies (those exhibiting overlapped binding footprint with ACE2) and non‐RBM antibodies (those not competing with ACE2 for RBD binding) in serum. This is based on the findings that the Omicron variant possessed a denser distribution of mutations in the apex RBM than in other regions (Figure 1A). As a result, antibodies recognizing the RBM also lost neutralization more pronouncedly than antibodies targeting the non‐RBM regions, some of which still maintain comparable efficacy against Omicron. Interestingly, the levels of non‐RBM antibodies in vaccine recipients were positively associated with their neutralizing activity against Omicron, while the correlation was not observed in RBM‐ or whole RBD‐binding antibodies. These findings suggested that the non‐RBM antibodies may provide broad immune protection against variants. More importantly, COVID‐19 vaccine recipients in the study have higher level of RBM‐binding antibodies, illustrating the reason why first generation of COVID‐19 vaccines based on wild‐type (WT) strain have reduced protective ability for people from variants infection. Taken together, binding potency or ACE2‐competing ability of antibodies to the RBD cannot predict the neutralization against Omicron. In contrast, the proportion of non‐RBM antibodies in all the RBD‐specific antibodies in plasma may be a useful parameter to indicate the antibody breadth and Omicron neutralizing activity.

**FIGURE 1:**
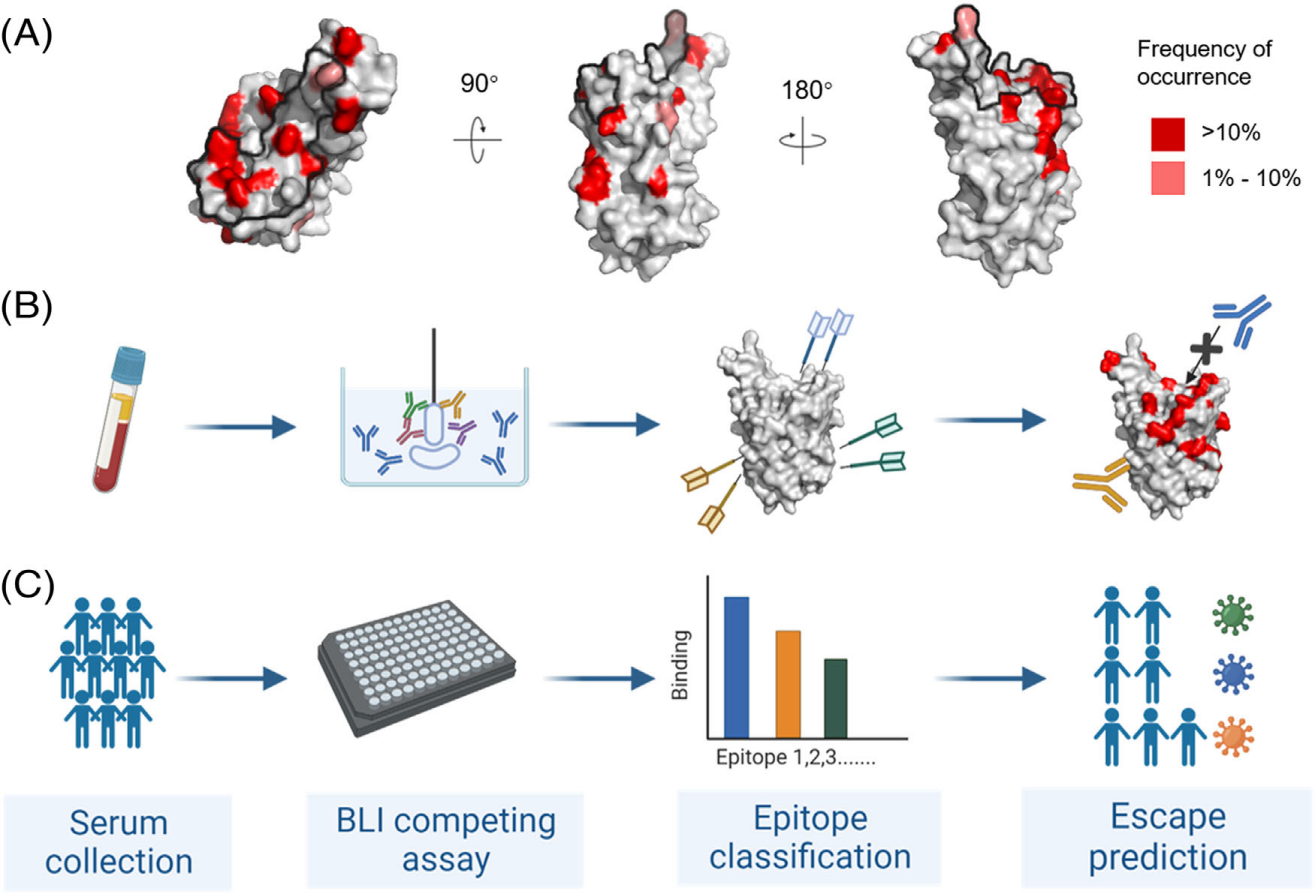
A denser distribution of mutations in the apex receptor‐binding motif (RBM) than in other regions (A), and the schematic of serum epitope mapping assay for prospective evaluation of its potency against new emerging SARS‐CoV‐2 variants (B and C). RBD from the top (left), outer side (middle) and inner side (right) views are shown as surface in grey (PDB 7E3O), with residues of mutation frequency more than 10% from public database (https://ngdc.cncb.ac.cn/ncov/variation/spike) highlighted in red, between 1% and 10% highlighted in salmon, and the ACE2‐binding site outlined by black line (A). The flowchart of serum epitope mapping by biolayer interferometry (BLI) assay and the flowing prospective evaluation against new variants based on mutation site analysis applied in a single serum sample (B) and as a high‐throughput method applied in a large population (C)

In addition to ACE2 competition, non‐RBM binding can be further subdivided into binding towards lateral surfaces facing the outside, hidden cryptic side, and inner cliff epitopes by competition assays using antibodies that bind to these epitopes, respectively. Interestingly, there is no mutation on the inner cryptic cliff surface of RBD by deep mutation analysis of existing SARS‐CoV‐2 variants. Antibodies targeting this site were cross‐reactive through all variants of concern (VOCs), even SARS‐CoV and other coronaviruses,^4^, ^5^ indicating that individuals with high serum antibody titres against this epitope are likely to show the broadest response to the existing variants.

Entering the fourth year of the COVID‐19 pandemic, we have witnessed the emergence and evolution of vast SARS‐CoV‐2 mutations and the intense escape of antibodies, causing difficulties in long‐term assessment of serum efficacy. The serological competition assay can be used to map the epitope landscape of antibodies in serum, and together with the mutation site distribution of viruses to predict the retention of antiviral activity of serum for a new variant (Figure 1B,C). Compared to samples with a higher diversity of antibody epitope distributions, serum binding to particularly concentrated epitopes are more likely to completely fail in neutralizing certain mutants. In addition, taxonomic large‐sample analysis of serum binding epitopes profile may also be used to monitor immunogenic epitopes in population, thereby estimating the antigenic evolution and possible emergence of new mutants under immune selection pressure, and guide the selection of effective vaccines.

## CONFLICT OF INTEREST

The authors declare they have no conflicts of interest.
